# Excimer laser treatment combined with riboflavin ultraviolet-A (UVA) collagen crosslinking (CXL) in keratoconus: a literature review

**DOI:** 10.1007/s10792-020-01394-5

**Published:** 2020-05-02

**Authors:** M. Ezzeldin, F. Filev, J. Steinberg, A. Frings

**Affiliations:** 1 Department of Ophthalmology, University Hospital Duesseldorf, Moorenstraße 5, 40225 Duesseldorf, Germany; 2 Department of Ophthalmology, University Hospital Hamburg-Eppendorf, Martinistraße 52, 20251 Hamburg, Germany; 3 Hamburg Visionclinic (Zentrum Sehstaerke), Martinistr. 64, 20251 Hamburg, Germany; 4 UCL Institute of Ophthalmology, 11-43 Bath St, London, EC1V 9EL UK; 5 Augenarztpraxis Dr. Frings, Färberstraße 20, 90402 Nuremberg, Germany

**Keywords:** Photorefractive keratectomy, Keratoconus, Collagen-crosslinking, Refractive surgery

## Abstract

**Purpose:**

To review the clinical outcome of keratoconus patients after excimer laser treatment with combined riboflavin UV-A collagen crosslinking (CXL) treatment was reviewed in light of the UDVA, CDVA and HOA.

**Methods:**

Following a PubMed-based literature review of studies on excimer laser treatment with combined riboflavin UV-A CXL published between 2009 and 2018, peer-reviewed English-written studies were evaluated using the GRADE approach (www.gradeworkinggroup.org). The current review focused on the change in the (un)corrected distance visual acuity (UDVA; CDVA) and higher-order aberrations (HOA) as well as the prevalence of postoperative complications.

**Results:**

Five studies with a total of 573 eyes were included, thereby reporting on 479 eyes were treated with the aforementioned combination therapy. The control group consisted of 94 eyes in total. Changes between pre- and postoperative CDVA and/or UDVA were statistically significant in all five studies after at least a 24-month follow-up period for the combined excimer laser-assisted CXL treatment in comparison with the CXL-only treatment option. Three studies described statistically significant reduction in the number of total HOA, in particular, those related to coma and spherical aberration. Corneal haze was reported in four studies, but the condition was successfully treated in all cases.

**Conclusion:**

Current studies suggest that CDVA, UDVA and HOA in low-to-moderate keratoconic patients improved in a combined treatment without sacrificing biomechanical stability of the cornea. However, long-term results are needed, as the studies in our review have a follow-up period of 68 months or less.

## Introduction

One of the current treatment options for keratoconus is a combined therapy of riboflavin ultraviolet-A (UVA) collagen crosslinking (CXL) which aims to stop disease progression and requires no lengthy and technically difficult surgical interventions.

Conventional CXL therapy performed in accordance with the Dresden Protocol requires the use of 0.1% riboflavin solution for 30 min prior to irradiation [2], after which UVA light is applied for an additional 30 min at an irradiance of 3 mW/cm^2^ with a total dose of 5.4 J/cm^2^. The 0.1% riboflavin solution is “reloaded” at a 5-min interval during the irradiation procedure. In accelerated CXL, higher UVA irradiation and shorter riboflavin intervals are applied, allowing for a much faster procedure in comparison with the “conventional” method. Despite these advantages, there is currently no standardized protocol for accelerated CXL, and the effectiveness of this treatment approach still remains controversial [3].

The many benefits of photorefractive keratectomy (PRK) make this therapy option distinctly advantageous to patients. No standardized guideline exists despite the variety of approaches that can be utilized for PRK, and more research is needed to understand the pros and cons of the associated techniques. To date, three studies were reported on topography-guided (TG-PRK), one looked at non-topography-guided, and one study was focused on wavefront-guided PRK. Each technique will be discussed in detail in the coming sections as appropriate.

Refractive surgeries have been considered to be a risk for patients with keratoconus, as it can lead to a progression of keratectasia [4, 5]. Therefore, there are up until today no common guidelines for the procedure of CXL in combination with any refractive surgery.

First published in 2009 [6], topography-guided photorefractive keratectomy (tPRK) with UVA-CXL was described as a new treatment option to maintain crosslinking stability and to improve visual acuity in keratoconus-afflicted patients, mainly by addressing higher-order aberrations (HOA) via a customized treatment approach [6].

In Kontadakis’ study, a TG-PRK was conducted with a 5.5-mm ablation zone in which correction customization was performed based on the corneal higher-order aberrations. Here, the correction percentage was individualized from 0% (conventional) to 100% (customized), and the 50 mm maximum ablation depth of the stromal thickness was still within the 400 mm safety limit. Alessio et al. performed a single-step, no-touch ablation transepithelial TG-PRK in which the corneal epithelium and stroma were removed to minimize tissue consumption. Here, the optical zone was between 2.16 and 5.45 mm, the transition zone diameter was between 6.00 and 8.75 mm, and the ablation stromal depth was between 18 and 19 mm (mean 31.1 ± 9.5). The selected epithelial thickness profile was 50 mm.

A WaveLight® Topolyzer was used by Kanellopoulos et al. to adjust the postoperative cornea asphericity to zero in all cases with no tilt, a transition zone of 1.5 mm, and a 5.5 mm optical zone (OZ). The optical zone was reduced to minimize tissue removal from the typical 6.5 mm zone in PRK and LASIK procedures. Stromal removal of 50 μm achieved ~ 70% treatment of the cylinder and up to 70% of the sphere. Al Amri utilized a Quest® excimer laser platform for non-topography-guided PRK.

Gore et al. focused on ocular wavefront-guided PRK in which the mean tissue diameter spared was 25 mm ± 16 (SD) in the thinnest part of the cornea (cone apex) when compared to corneal wavefront-guided treatments. In the ocular wavefront-guided PRK, the mean transepithelial ablation diameter was 8.73 ± 0.53 mm, with a mean OZ of 7.18 ± 0.58 mm.

This literature review was set up to summarize published studies on the role of excimer laser treatment combined with UVA-CXL to give readers an overview of current indications, results and limitations of a treatment option that has gained popularity in recent times.

Review as by Al-Mohaimeed [7] et al. and Zhu et al. [8] looked at comparing various adjuvant treatments, such as intrastromal ring segments, conductive keratoplasty or phakic intraocular lens, and discussed the associated safety and efficacy of these therapies. Here, it was established that combined refractive surgery therapies such as the PRK-CXL approach resulted in only a few minor complications and was effective at halting the progression of keratoconus. In our review, the use of PRK treatment combined with CXL/ accelerated CXL is discussed in light of the absolute outcomes of CDVA, UDVA and HOA as a summary of various published studies on the role of excimer laser-assisted treatment combined with UVA-CXL. This review gives readers an overview of the current indications, results and limitations of a treatment option that has gained popularity in recent times.

## Methods

In January 2019, we performed a literature review via PubMed using the keywords “photorefractive keratectomy,” “collagen crosslinking” and “keratoconus,” and their commonly accepted acronyms. To grant an eligible comparison between the studies, only peer-reviewed studies published in English were selected for further analysis. We excluded studies with a follow-up (FU) of less than 24 months.

**Fig. 1:**
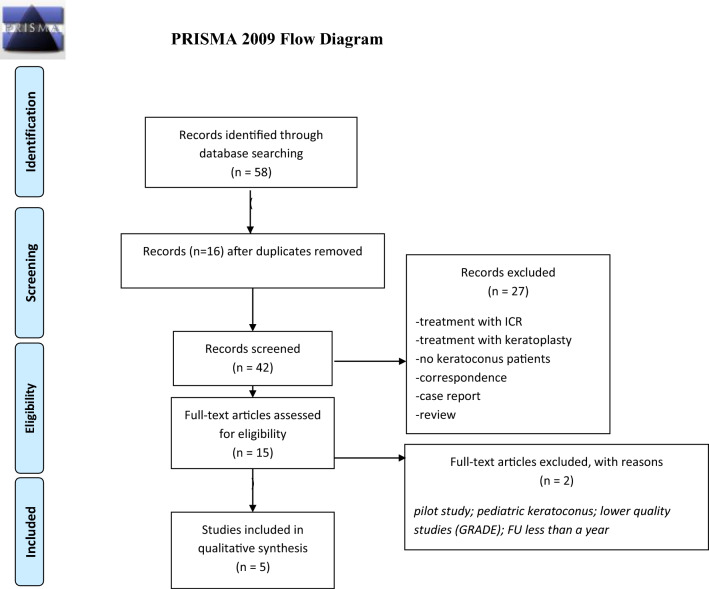
Applied screening process of the literature to grant an eligible comparison between the studies

To evaluate the scientific quality of the given studies, the Grading of Recommendation Assessment, Development and Evaluation (GRADE) approach to evalutate their scientific quality and appropriateness for our review. The assessment was conducted by three independent readers (ME, FF, AF). The evaluation scheme can be found in Table 1, whereas Fig. 1 summarizes the screening process.

**Table 1:** Quality assessment following the grading of recommendation assessment, development and evaluation (GRADE) approach

Name of the published study	Quality of evidence (GRADE)
Combined wavefront-guided transepithelial photorefractive keratectomy and corneal crosslinking for visual rehabilitation in moderate keratoconus	⊕ ⊕ ⊕ ⊕ High
5-year follow-up of combined nontopography-guided photorefractive keratectomy and corneal collagen crosslinking for keratoconus	⊕ ⊕ ⊕ ⊕ High
Long-term comparison of simultaneous topography-guided photorefractive keratectomy followed by corneal crosslinking versus corneal crosslinking alone	⊕ ⊕ ⊕ ⊕ High
Photorefractive keratectomy followed by crosslinking versus crosslinking alone for management of progressive keratoconus: two- year follow-up	⊕ ⊕ ⊕ ⊕ High
Comparison of sequential versus same-day simultaneous collagen crosslinking and topography-guided PRK for treatment of keratoconus	⊕ ⊕ ⊕ ⊕ High

As the primary outcome parameter of our shortlisted studies, the uncorrected (UDVA) and corrected (CDVA) distance visual acuity at last FU was compared to baseline (Tables. 3 and 4) and the changes noted in HOA were examined where given.

In studies where patients were tested more than once after the intervention, only the last FU data were reported.

## Results

Between September 2009 and December 2018, five studies were identified that matched the inclusion criteria (Table 1 and Fig. 1). These studies were conducted in Greece, the USA, the UK, Italy and Saudi Arabia, and were published in five different medical journals (Table 2).

**Table 2:** Published studies included in this review, listed by year of publication

Title of study	Year of publication	Published in	Study design	Number of eyes included	Age at treatment (mean ± standard deviation, range)	Stage of keratokonus
Combined wavefront-guided transepithelial photorefractive keratectomy and corneal crosslinking for visual rehabilitation in moderate keratoconus	2018	J Cataract Refract Surg	Prospective, controlled, case series	94	24.6 ± 3.8 (18–35)	All eyes: stage 1–3 according to Amsler Krumeich
5-year follow-up of combined nontopography-guided photorefractive keratectomy and corneal collagen crosslinking for keratoconus	2018	Int J Ophthalmol	Prospective, non-controlled case series	60	28 ± 5.4 (21–41)	All eyes: stage 1–2 according to Amsler Krumeich
Long-term comparison of simultaneous topography-guided photorefractive keratectomy followed by corneal crosslinking versus corneal crosslinking alone	2016	Ophthalmology; 123:974–983	Prospective, controlled, interventional case series	60	28 ± 5.82 (17–39) CXL group: 28 ± 3.99 (17–39) tPRK-CXL: 27 ± 7.24 (17–39)	CXL group according to Amsler Krumeich: 10 eyes stage 1; 17 eyes stage 2; 3 eyes stage 3 tPRK plus CXL according to Amsler Krumeich: 14 eyes stage 1; 14 eyes stage 2; 2 eyes stage 3
Photorefractive keratectomy followed by crosslinking versus crosslinking alone for management of progressive keratoconus: 2-year follow-up	2013	Am J Ophthalmol	Prospective, controlled, interventional, case series	34	31.17 ± 8.12 (21–46)	PRK + CXL: 9 eyes stage 1; 5 eyes stage 2; 3 eyes stage 3 CXL: 10 eyes stage 1; 4 eyes stage 2; 3 eyes stage 3 According to Alió and Shabayek classification [14]
Comparison of sequential versus same-day Simultaneous collagen crosslinking and topography-guided PRK for treatment of keratoconus	2009	J Refract Surg.;25:S812S818	Retrospective, controlled, case series	325	CXL, 6 month later PRK 21.5 SD not given (17–29) CXL and PRK on the same day 20.5 SD not given(16–29)	Progressive keratoconus = corneal steepening of 1 diopter keratometry and increasing myopia and /or astigmatism over 3 months

Title of study	Type of treatment	CXL protocol	Control group	FU (months)	CDVA at last FU in logMAR
Combined wavefront-guided transepithelial photorefractive keratectomy and corneal crosslinking for visual rehabilitation in moderate keratoconus	Wavefront-guided tPRK plus accelerated CXL or accelerated CXL alone	0.1% riboflavin, in total 10 min; 8 min at an irradiance of 30.0 mW/cm^2^ (7.2 J/ cm^2^)	Yes	24	tPRK + accelerated CXL: 0.15 ± 0.14 CXL: 0.17
5-year follow-up of combined nontopography-guided photorefractive keratectomy and corneal collagen crosslinking for keratoconus	Simultaneous nontopography-guided PRK and CXL	0.1% riboflavin for 30 min; 15 min at an irradiance of 3.0 mW/cm^2^; Continuous riboflavin application during UVA every 2 min	No	60	0.03 ± 0.12
Long-term comparison of simultaneous topography-guided photorefractive keratectomy followed by corneal crosslinking versus corneal crosslinking alone	tPRK followed by CXL or CXL alone	0.1% riboflavin, 30 min; 30 min at an irradiance of 3.0 mW/cm^2^	Yes	39 ± 11	CXL: 0.15 ± 0.12 tPRK-CXL: 0.09 ± 0.10
Photorefractive keratectomy followed by crosslinking versus crosslinking alone for management of progressive keratoconus: 2-year follow-up	PRK + CXL CXL alone	Riboflavin 0.1%, 20 min; continuous riboflavin application during CXL treatment	Yes	24	PRK + CXL: 0.03 ± 0.06 CXL:0.04 ± 0.07
Comparison of sequential versus same-day Simultaneous collagen crosslinking and topography-guided PRK for treatment of keratoconus	CXL + 6 months later topography-guided PRK CXL and same day topography-guided PRK	0.1% riboflavin, 10 min, every 2 min; 30 min at an irradiance of 3 mW/cm^2^	Yes	24–68	Sequential CXL and PRK: 0.16 ± 0.22 Simultaneous CXL and PRK: 0.11 ± 0.16

*CDVA* corrected distance visual acuity, *CXL* corneal collagen crosslinking, *FU* follow-up, *tPRK* transepithelial photorefractive keratectomy, *tPTK* transepithelial phototherapeutic keratectomy

In all studies, patients underwent a full ophthalmological examination, and the study protocols employed were adequately described for replication. Additionally, various study designs were used, including a prospective controlled study (*n* = 3) [9–11], a prospective non-controlled study (*n* = 1) [12] and a retrospective controlled study (*n* = 1) [6] with a relatively large number of examined eyes. The FU period in our shortlisted studies ranged from 24 to 68 months, and the study cohorts were between 34 [11] and 325 [6] individuals. The study group with the youngest mean age of 24.6 ± 3.8 years was reported by Gore et al. [9]. Further patient characteristics and the relevant refractive data are summarized in Tables 2 and 3.

**Table 3:** Summary of pre- and postoperative (un)corrected distance visual acuity (in logMAR)

Study	Types of treatment	Preop mean CDVA ± standard deviation	Postop mean CDVA ± standard deviation	Preop mean UDVA ± standard deviation	Postop mean UDVA ± standard deviation
Gore et al. [9]	tPRK + accelerated CXL	0.28 ± 0.21	0.15 ± 0.14	0.72	0.52
	CXL	0.22 ± 0.13	0.22 ± 0.13	0.63	0.66
Kontadakis et al. [10]	tPRK + CXL	0.26 ± 0.17	0.09 ± 0.10	0.83 ± 0.54	0.27 ± 0.25
	CXL	0.24 ± 0.18	0.15 ± 0.12	0.86 ± 0.62	0.69 ± 0.58
Kanellopoulos [6]	PRK + CXL (sequential)	0.41 ± 0.25	0.16 ± 0.22	0.9 ± 0.3	0.49 ± 0.25
	PRK + CXL (simultaneous)	0.39 ± 0.3	0.11 ± 0.16	0.96 ± 0.2	0.3 ± 0.2
Alessio et al. [11]	PRK + CXL	0.06 ± 0.08	0.03 ± 0.06	0.63 ± 0.36	0.19 ± 0.17
	CXL	0.06 ± 0.11	0.04 ± 0.007	0.59 ± 0.29	0.52 ± 0.29
Al Amri [12]	Non-topography-guided PRK + CXL	0.06 ± 0.19	0.03 ± 0.12	1.24 ± 0.79	0.06 ± 0.15

*PRK* photorefractive keratectomy, *tPRK* transepithelial photorefractive keratectomy, *CXL* collagen crosslinking, *CDVA* corrected distance visual aquity, *UDVA* uncorrected distance visual aquity

The different types of adjuvant therapies that involve the use of excimer laser treatment with CXL are summarized in Table 2. Thus, the five shortlisted studies can be further categorized according to the type of PRK therapy employed, namely wavefront-guided (*n* = 1) [9], non-topography-guided (*n* = 1) [12] and topography-guided PRK (*n* = 3) [6, 10, 11]. Moreover, the classification of the keratoconic condition in the patients in each study is detailed in Table 2.

Pre- and postoperative UDVA and CDVA are summarized for each study (Table 3). All five studies presented data that demonstrated a statistically significant gain of two lines or more in CDVA (Table  3).

Two studies reported on the changes observed in HOA (Table 4). Here, all outcomes revealed a statistically significant improvement in total HOA after the application of the respective combined treatment option.

**Table 4:** Summary of pre- and postoperative HOA: total higher-order aberrations, SA: spherical aberration

Study	Pupil size diameter (mm)	Preop mean HOA (RMS) ± standard deviation (SD) (µm)	Postop mean HOA (RMS) ± SD (µm)	Preop mean coma ± SD (µm)	Postop mean coma ± SD (µm)	Preop mean SA ± SD (µm)	Postop mean SA ± SD (µm)	Preop mean trefoil ± SD (µm)	Postop mean trefoil ± SD (µm)
Gore et al. [9]									
Trans PRK/CXL	6	2.36 ± 0.74	1.20 ± 0.65	1.96 ± 0.8	0.87 ± 0.63	0.71 ± 0.53	0.32 ± 0.35	0.44 ± 0.32	0.41 ± 0.25
		*P* < 0.001		*P* < 0.001		*P* < 0.001		*P* = 0.634	
CXL	6	2.66 ± 0.96	2.54 ± 0.91	2.40 ± 0.97	2.27 ± 0.92	0.48 ± 0.47	0.46 ± 0.54	0.42 ± 0.26	0.45 ± 0.27
		*P* = 0.507		*P* = 0.489		*P* = 0.857		*P* = 0.578	
Alessio et al. [11]									
PRK + CXL	3	1.94 ± 0.62	1.14 ± 0.60	1.4 ± 5 0.60	0.81 ± 0.51	–	–	–	–
		*P* < 0.001		*P* < 0.001					
	5	5.57 ± 1.90	3.44 ± 1.76	4.76 ± 2.00	2.75 ± 1.64	–	–	–	–
		*P* < 0.001		*P* < 0.001					
	7	10.66 ± 3.82	7.19 ± 3.75	9.27 ± 3.90	5.60 ± 3.82	–	–	–	–
		*P* < 0.001		*P* < 0.001					
CXL	3	1.79 ± 0.81	1.68 ± 0.86	1.42 ± 0.56	1.30 ± 0.50	–	–	–	–
		*P* = 0.325		*P* = 0.052					
	5	5.76 ± 1.99	5.12 ± 1.97	4.70 ± 1.66	4.41 ± 1.57	–	–	–	–
		*P* = 0.093		*P* = 0.073					
	7	11.18 ± 3.83	10.50 ± 3.70	9.41 ± 3.73	9.20 ± 4.03	–	–	–	–
		*P* = 0.141		*P* = 0.355					

In four studies, minor complications were described (i.e., haze formation), which were either self-limiting until the last FU or treated successfully [6, 9, 11, 12].

## Discussion

The findings of this literature review suggest that the biomechanical stability of the combined (t)PRK-CXL treatment approach is on par with that of the CXL-only treatment option. In both cases, the progression of keratoconus was effectively halted within the FU timeframe presented. It seems to be as safe as a CXL treatment alone in terms of biomechanical stability. However, this is only valid for the FU as presented in the analyzed studies. Further the adjuvant therapy leads to an enhanced efficacy by improving CDVA, mainly by addressing HOA [11].

In our review, we were able to include five remaining studies after applying the above-mentioned criteria. The analyzed studies report on treatment results after different types of CXL—conventional or accelerated—and different excimer laser treatment approaches. If the studies presented a control group, the patients were treated with accelerated or conventional CXL only.

In three of the studies reported, a statistically significant improvement in CDVA was noted after excimer laser treatment combined with CXL [9, 10, 12]. Furthermore, there were reports on the significant improvement observed in the UDVA values [10–12], which were attributed to a reduction in HOAs, especially coma, which should be defined as the main goal when treating eyes with keratoconus by excimer laser surgery. Kontadakis et al. [10] achieved up to 33% correction of refractive myopia and up to 66% correction of the refractive cylinder. Nevertheless, when treating keratoconic patients, we must not strive to treat lower-order aberrations to the levels of full refractive vision correction typically seen in healthy candidates [9].

Special considerations when planning the CXL treatment should be noted. This point was of particular concern to Gore et al. [9] since it was discovered that the laser treatment-induced deeper ablation of the Bowman membrane, which led to a more efficient absorption of riboflavin and UVA, resulting in more extensive CXL effect. Kampik et al. pointed out that the parameters for excimer laser treatment must be continuously adjusted in light of a “stiffening” effect often induced when laser-assisted treatment and CXL were performed consecutively [13]. Kanellopoulos et al. showed that same-day simultaneously topography-guided PRK and CXL lead to statistically significantly (*P* < 0.05) better results compared to sequential CXL with a consecutive PRK therapy. Here, UDVA and CDVA exhibited more notable reduction in the spherical equivalent and less corneal haze [6]. The authors of that study theorized that simultaneous topography-guided PRK and CXL appeared to be more effective due to either better penetration of the riboflavin solution through the ablated stroma or the absence of the Bowman’s layer. Moreover, it was suggested that the corneal shape was more resistant to the progression of the disease [6]. Unfortunately, whether adjuvant treatment should be applied simultaneously or sequentially was not an issue that was evaluated in the other studies and must be addressed in future trials.

As a possible side effect Kampik et al. [13] raise caution that the cornea could be damaged by higher UVA exposure as the Bowman membrane is ablated and haze could appear more easily. Haze usually decreases in the first postoperative year. Haze after CXL can be differentiated from haze after PRK by stromal depth. Whereas haze after PRK usually is subepithelial, haze after CXL extends into the anterior stroma to approximately 60% depth, approximately 300 µm in an average cornea. The origin of post-CXL haze is associated with loss of keratocytes [13].

Other than the scant number of studies that served as the basis of this review, the main limitations were the dearth of randomized studies and the vague definition of what entails progressive keratoconus. Here, the term is defined depending on the guidelines being used by the respective authors, as these can vary from the Amsler Krumeich classification to an increase in “cone apex of 0.75 D” or a myopic shift of 0.75 D.

In the studies presented here, most patients demonstrated a keratoconus stage according to Amsler Krumeich of 3 or less, and all studies mentioned herein were comprised of patients with an average corneal thickness of at least 350 μm. Therefore, treatment was only attempted in less progressive, mild-to-moderate cases of keratoconus. Unfortunately, the lack of long-term study results with lengthy FU periods is also a limiting factor in the current review.

In conclusion, current literature shows evidence that a combined (t)PRK-CXL therapy is an efficient and safe method for stabilizing the cornea of mild-to-moderate keratoconus eyes. Here, the occurrence of HOA was reduced through improved CDVA values. It is still not clear if this approach was more effective or as safe as the use of CXL-only treatment options, particularly when comparing the long-term outcome concerning corneal biomechanics. Additional prospective randomized studies with long-term observations, biomechanical long-term examinations and other subjective outcome measurements are needed to conduct a thorough evaluation of the safety, efficacy and predictability of this treatment approach.
